# Type 2 diabetes management: Patient knowledge and health care team perceptions, South Africa

**DOI:** 10.4102/phcfm.v4i1.392

**Published:** 2012-10-18

**Authors:** Nombeko Mshunqane, Aimee V. Stewart, Allan D. Rothberg

**Affiliations:** 1 Department of Basic Medical Sciences, Durban University of Technology, South Africa; 2 Department of Physiotherapy, University of the Witwatersrand, South Africa

## Abstract

**Background:**

South African research indicates that the highest death rates between 2004 and 2005 were from diabetes mellitus. There is minimal research information on interactions between what patients know about their disease and what health professionals perceive that patients should know to control their disease well.

**Objectives:**

This study determined the knowledge that patients with type 2 diabetes have about the management of their disease, as well as the perceptions of the health care team about the services given to patients.

**Method:**

Qualitative data were collected using two focus groups and in-depth interviews. Patient focus group (*n* = 10) explored patients’ knowledge about management of type 2 diabetes. Patients were recruited from Dr George Mukhari Hospital outpatients’ diabetes clinic. Professional focus group (*n* = 8) explored the health care team's experiences, barriers and facilitators in managing the disease. Professional focus group participants were recruited because of their expertise in chronic disease management, working in the community (public health) or working directly with patients with type 2 diabetes. Five health care professionals were interviewed using the same guide of questions as for the focus group.

**Results:**

Participants identified type 2 diabetes as a chronic disease that needs behaviour change for good control. Five major themes were identified: patients’ knowledge; education programmes; behaviour change; support; and a patient-centred approach.

**Conclusion:**

Management of type 2 diabetes may be enhanced by reinforcing patients’ knowledge, encouraging behaviour change whilst taking into consideration patients’ backgrounds. The health care team needs to utilise a patient-centred approach.

## Abstrait

**Prise en charge des diabètes de type 2: Connaissance des patients et perception des équipes de soins de santé, Afrique du Sud**

**Contexte:**

La recherche sud-africaine révèle que les plus hauts taux de mortalité entre 2004 et 2005 étaient dus au diabète mellitus. Il y a très peu d'information sur les interactions entre ce que les patients savent de leur maladie et ce que les professionnels de la santé perçoivent des connaissances que les patients devraient avoir pour bien contrôler leur maladie.

**Objectifs:**

Cette étude a permis de déterminer les connaissances des patients atteints de diabète de type 2 sur la prise en charge de leur maladie, ainsi que la perception de l’équipe de soins de santé sur les services fournis aux patients.

**Méthode:**

Des données qualitatives ont été recueillies par le biais de deux groupes de discussion et d'entretiens approfondis. Le groupe de discussion des patients (*n* = 10) a permis d’étudier les connaissances des patients sur la prise en charge du diabète de type 2. Les patients ont été recrutés parmi les patients externes de la clinique du diabète à l'hôpital George Mukhari. Le groupe de discussion des professionnels (*n* = 8) a permis d’étudier les expériences, obstacles et facilitateurs en matière de prise en charge de la maladie de l’équipe de soins de santé. Les participants au groupe de discussion des professionnels ont été recrutés pour leur expertise en matière de prise en charge de maladies chroniques, qu'ils travaillent dans les communautés (santé publique) ou directement auprès de patients atteints de diabète de type 2. Cinq professionnels des soins de santé ont été interrogés en utilisant la même grille de questions que celle du groupe de discussion.

**Résultats:**

Les participants ont identifié le diabète de type 2 comme une maladie chronique qui demande des changements de comportements pour un bon contrôle de la maladie. Cinq grands thèmes ont été retenus: la connaissance des patients; les programmes d’éducation; le changement de comportement ; le soutien et l'approche axée sur le patient.

**Conclusion:**

La prise en charge du diabète de type 2 peut être améliorée en renforçant les connaissances des patients, en encourageant un changement de comportement tout en prenant en considération les antécédents des patients. L’équipe de soins de santé doit utiliser une approche axée sur le patient.

## Introduction

### Key focus

The prevalence of type 2 diabetes continues to increase, especially in developing countries, despite improvements in research.^1^ Two focus groups, one involving patients and the other health professionals, identified five similar themes around knowledge and management of type 2 diabetes. Whilst themes were similar, it was clear that behaviour change was affected by environmental factors and lack of education programmes for patients to understand the disease process. This was influenced by a lack of resources in the public sector. Failure to address these factors is likely to result in sub-optimal disease management.

#### Background and trends

Type 2 diabetes is one of the most common chronic diseases which are influenced by the lifestyle of an individual.^2^ It is a quiet killer,^3^ constituting 37% of the mortality in 2000; 36% of deaths in males and 40% in females.^4, 5^ The prevalence of the disease continues to increase worldwide, especially in developing regions.^6^ The prevalence rates suggest that increasing westernisation and urbanisation are responsible.^4–6^ The disease not only affects patients but also places a burden on the world health systems’ economy.^6^ In 2004 it was estimated that there were a million patients diagnosed with diabetes, with possibly the same number of cases undiagnosed, in South Africa alone.^5^

Abnormal beta cell function results in relative insulin deficiency and hyperglycaemia.^7^ Hyperglycaemia results in micro- and macrovascular complications.^8, 9^ These complications result in a negative economic impact, which can be delayed by prevention programmes.^10, 11^ In both type 1 and type 2 diabetes mellitus hyperglycaemia is better controlled if the patient participates in regular exercise.^12, 13^ The complications account for a large percentage of non-traumatic amputation of the lower limbs, ischaemic heart disease, blindness and end-stage kidney disease.^12, 14^

Management of this disease is aimed at helping patients and their families to gain knowledge, skills, resources and support essential for optimal health.^9^ A team effort of health care professionals combined with patients is therefore crucial.^1, 9^ The United Nations and International Diabetes Federation adopted a resolution to stop the growing epidemic of diabetes, where the main focus of their action plan was to encourage government to implement actions to educate and inform populations about primary and secondary diabetes prevention, treatment and care.^6^

#### Objectives

The main purpose of this study was to determine the knowledge of patients with type 2 diabetes about their management. The study also determined the perceptions of the health care team about the management given to patients with type 2 diabetes, looking at barriers and facilitators.

#### Contribution to field

By highlighting the existing barriers to or facilitators of the management of type 2 diabetes as viewed by patients and health care professionals, a need to improve the quality of care in South African public hospitals may be revealed. These results would enlighten the Department of Health of the need to develop and implement strategies to improve quality of care in type 2 diabetes management. On the other hand, if the existing standards of care are perceived as good by patients and the health care team, the Department of Health will also be informed of good quality of care practice.

## Ethical considerations

Consent was obtained from all participants, and they were aware that all discussions were tape-recorded. Ethical clearance was obtained from the University of the Witwatersrand, Johannesburg, South Africa, clearance number M060955. A signed consent form was obtained from each participant who volunteered to take part in the study.

### Potential benefits and hazards

The study had no envisaged hazards to the study population. Benefits included enlightening the health care providers about the knowledge the patients had about the management of type 2 diabetes, as well as challenges they experienced which limited them in achieving optimal treatment goals. All data collection sheets and tapes were kept confidential by the researcher.

### Recruitment procedures

All participants volunteered to take part in this study and gave their consent. No incentives were provided. Participants were allowed to withdraw at any time if they wanted. All patients were recruited from Dr George Mukhari Hospital outpatients’ diabetes clinic. Participants in the professional focus group were invited telephonically followed by an email invitation, and were from the three medical universities in Gauteng Province, including those working at Dr George Mukhari Hospital and community centres around the hospital.

### Informed consent

The procedure was explained to all participants including the use of a tape-recorder throughout the discussion, using the information sheet. Participants were then asked to sign a consent form upon agreeing to take part in the study.

### Data storage

All participants were informed that the data collection sheets would be stored in the researcher's office and that the information would only be used for this research.

## Methods

### Materials

The format for the design and use of focus groups was followed when conducting this study.^15, 16^ Two focus groups were conducted to answer the above objectives. Both discussions were carefully planned and designed to obtain perceptions in a permissive, non-threatening environment. The interview was guided by a moderator.^16^ Questions were open-ended and enough time was allowed for comments. Questions were more general at the beginning of the session and became more specific and focused as the session continued.^17–19^ The patient focus group consisted of 10 participants and the professional focus group had eight participants.^16^

### Setting

The study was conducted at Dr George Mukhari Hospital, which is an academic hospital for the University of Limpopo, Medunsa Campus, formerly known as MEDUNSA (Medical University of Southern Africa). It is the second largest referral hospital in South Africa with 1550 beds. It provides services for patients from seven of the surrounding townships: Mabopane, Soshanguve, Mamelodi, Atteridgeville, Temba, Lethlabile including Ga–Rankuwa which has 14 zones, and Winterveld, which is a rural area.

### Design

A cross-sectional survey with a qualitative approach was undertaken.

### Procedure

#### Selection of focus group participants

Selection criteria for the study included patients who had been diagnosed with type 2 diabetes for at least one year, who were between 30 and 65 years of age (patient focus group). Participants for this group were recruited from the outpatients’ diabetes clinic at Dr George Mukhari Hospital after a 20-minute talk about diabetes. Twenty patients with type 2 diabetes volunteered to participate and gave their telephone numbers for follow-up reminders. Only 10 participants were randomly selected to participate in the study. Health care professionals were selected based on their expertise in chronic disease management, working in community settings (public health) or directly with patients with type 2 diabetes. One moderator for each group was identified.^15, 16^

#### Formulation of questions

Questions for the two focus groups were formulated according to the reviewed literature on focus group discussions, so that the purpose and objectives of the study were addressed.^17–19^ The interview questions were designed so that the language was easy to understand, because most participants were not first-language English speakers. Questions for both focus groups were pre-tested (for validity) on a sample that did not participate in the study.^15, 16^

#### Description of focus group

The patient focus group included 10 patients with type 2 diabetes, whilst the professional focus group included eight health care professionals working with patients with diabetes or public health workers. The discussion lasted for an hour and a half. A moderator and a research assistant were present in both groups. Both focus group discussions were tape-recorded. A free-flowing discussion was encouraged and the moderator followed a pre-planned script of questions. In-depth interviews were conducted with health care professionals who could not attend the focus group for various reasons. These interviews were also tape-recorded and field notes were taken.

### Analysis

All recorded information was transcribed at the end of all interviews by three different people. The researcher checked that the transcripts corresponded with field notes that were taken during the interviews. Authenticity of the transcripts was verified by giving the transcripts back to the participants for checking. Two participants from both focus groups verified the transcripts. Transcripts of individual interviews were given back to each participant to check. No changes were made to any of the transcripts. The data were categorised into concepts and thereafter into categories, using axial coding, and the categories were grouped into various themes.^16, 20^ Themes were developed from each focus group following all the responses through using a vertical and horizontal approach, across the groups.

An independent coder who was familiar with qualitative research was appointed to confirm whether coding was done properly and to confirm the themes (the coder was not involved in the focus group discussions). Information from the two focus groups’ responses and interviews was provided. A meeting was arranged with the coder to discuss the themes, and there was 90% agreement on the themes that were developed.^17, 19^

## Results

The responses for both focus groups are presented using the format described in the health belief model (Table 1).^21^

**TABLE 1:** Responses from the patient and professional focus discussions using the health belief model.

Concept	Definition: Type 2 Diabetes	Application
**Perceived susceptibility**	Patients mentioned pregnancy; hereditary factors; stress; obesity; unhealthy eating; physical inactivity; and hypertension as causes. Professional focus group felt that Westernisation, physical inactivity and uncontrolled food portions increase the prevalence of type 2 diabetes.	Affects both males and females between ages of 30–65 years. Professionals felt that Westernisation affects both young and old (20–65 years). Patients have to understand that the disease is a metabolic syndrome (theme: knowledge).
**Perceived severity**	Patients believe that the consequences of increased blood sugar levels are significant enough to cause complications and they should be avoided, that is avoid getting angry, avoid heaters (non-healing wounds), avoid sweets, avoid fatty foods. Professionals felt that patients do not have an understanding of the disease despite the talks that are given to them. They believe that patients consult other sources before Western medicine (e.g. traditional healers) because they are looking for a cure.	They believe that if the disease is not controlled by medication and behaviour change, this will lead to development of complications. Patients should understand that there is no cure but the disease can only be controlled by medication, exercise and following a proper diet (theme: behaviour change).
**Perceived benefits**	Patients believe that eating healthily, exercises, and taking your medication as prescribed as well as having family support will delay disease complications. Professionals believed that weight loss, lifestyle modification and informational support by educating the spouses was necessary as African men do not cook. Patients also need material support as many of the patients miss their appointments because they lack funds for transport. They also need emotional support from family and friends.	Understanding that there is no cure for the disease, but it can be controlled. There is a need to educate the person preparing food, friends and family (theme: support).
**Perceived barriers**	Patients identified their main barriers as acceptance, socio-economic status and avoidance by people around them (community). They always ask themselves the following questions after the diagnosis: Why me? What am I going to eat? What are the people going to say about me? Where will I get money to buy food? Professionals believed that patients’ barriers included cultural issues, limited resources in public sector, attitudes towards health professionals, poor adherence and socio-economic issues.	As individuals, they see the disease as a death sentence and a stigma, especially losing weight given the HIV epidemic. Support makes them feel better. Women would be resistant to losing weight because they want to maintain their image. Limited resources make doctors cut down the contact time with patients and they do not listen to patients’ problems; this creates poor attitudes towards health professionals. Because the disease is a syndrome, patients do not understand why they should take their medication regularly when they do not feel sick (theme: education).
**Cues to action**		
	Patients believe that if they can be empowered more through educational programmes (radio/television), control can be better. They feel that there are fewer programmes on diabetes awareness. Professionals believe that type 2 diabetes management should be nurse-based, there should be more public education and patients should be told the truth about disease management and availability of human resource.	Diabetes needs to be treated like HIV –more knowledge should be given to people through campaigns and television shows. Theme: patient-centred approach.
**Self-efficacy**	Patients believe that change of behaviour is important for better control. Professionals believe there must be more awareness about the disease and that understanding how the patient feels is important.	They are confident that they will engage themselves in exercises and change their behaviors about food with support. Nurse-based intervention.

## Discussion

### Outline of the themes

#### Health communication (knowledge)

Chronic diseases of lifestyle require a sense of coherent management, where a patient, the doctor, family and community work together to manage the disease. Patients’ education should aim at enhancing personal control over day-to-day management in a way that will improve their quality of life, rather than focusing on curing the disease.^22^ This means that government, communities, service providers and patients with type 2 diabetes should work together to manage the disease. Successful chronic disease management is dependent on effective, systematic and interactive communication between patients and service providers as well as the health system with which they make contact. This approach focuses on primary care by improving communication between patient and physician as well as identifying environmental problems from the patient's perspective.^23, 24^

Knowledge emerged as the main problem in the management of type 2 diabetes by both patients and health care professionals. Patients indicated the importance of knowledge in how they interpreted the diagnosis of type 2 diabetes.^25, 26^ According to the health care professionals in this study, the knowledge component of type 2 diabetes management included participants’ understanding of types of food, food portions and appropriate times that food should be eaten. In this study some patients knew about the recommended food practices, but because of socio-economic barriers (lack of finances) were unable to acquire the right kind of food. Some of the challenges to dietary adherence involve avoiding favourite foods, selecting healthful alternatives, time management (patients find it difficult to plan food with insulin or oral medication) and social support (as most women prepare food for their families).^26^ Patients need to eat the right amount of food for their normal body mass, that is high in starch and fibre but low in saturated fats (there is still a gap as to what is the correct diet).^8, 13^ They need to engage in moderate-low endurance type of exercises for plus or minus 20–30 minutes three or four times a week in order to improve their cardiovascular health.^9, 12^ Weight loss and adherence to their prescribed medication will also help to improve glycaemic control.^6, 12, 27^

In this study patients showed an understanding of the causes and complications of the disease, but most of them understood these negatively (as fears) – as confirmed by participant 6 when asked about the reaction when diagnosed with diabetes: ‘A death sentence, you think of the complications’. Participant 10, a 30-year-old male, when thinking about impotence, said: ‘My family is gone.’ They lacked a sense of positive thinking, namely that managing the disease well with good control will delay the onset of complications. It is therefore clear that to improve quality of care in this population, a collaborative model of chronic disease management which includes education and support should be developed.

The professionals felt that patients should be encouraged to understand the meaning of chronic disease and its management as referring to medical interventions that can only control but not cure the disease.^28^ They also emphasised that patients should know that the disease is a syndrome, which means they need to treat the disease continuously even if they do not feel sick, as confirmed by participant 1: ‘Patients always come to consult medical help when complications set in because they say: “why do I have to take medication even though I don't feel sick?”’ This typifies a barrier to adherence to their management, and leads to poor disease control.

#### Education

According to Glanz, Lewis and Rimer (1997), ‘Health education is the process of assisting individuals, acting separately or collectively, to make informed decisions about matters affecting their personal health and those of others’.^23^ Health behaviour is further described as referring to the actions of individuals, groups, and organisations and to those actions’ determinants, correlates and consequences, including social change, policy development and implementation.^23, 25, 29^ This means that health talks to patients should be goal-directed and must not only address the individual but the people around them as well.

In this study patients emphasised that education programmes should be more public, just like HIV education programmes. This was confirmed by participant 4, who asked: ‘What are people going to say about me when I lose weight?’ They went on to say that one only knows about diabetes when one is diagnosed or there is someone with diabetes at home. Professionals emphasised that diabetes education should be nurse-based in order to prevent complications. Professionals also added that patients should be told the truth about the lack of human resources in the public sector. This lack of human resources results in patients being given short consultation times and having long waiting times. This may have a negative impact on patients’ attitudes. This was confirmed by participant 4: ‘We should explain to patients and listen more; don't be judgmental’.

It is therefore important that behaviour should be evaluated individually, and the health belief model helps to bring clarity to individual health behaviour.^21^ An impact can only be made on behaviour when the patient has a good understanding of the disease process (informational approach) as well as disease management. This was confirmed by patient participant 3: ‘At work we were supplied with a “finger lunch” every time we have a meeting, now I have to change the way I eat – diabetic food is expensive.’ Patients need to be encouraged to actively participate so as to ‘voice out’ their fear and strengths about the management of their disease.^9, 12, 29^ Interestingly, in the professional group and in-depth interviews cultural issues were considered to be the most challenging barrier to management of type 2 diabetes; for example, the fact that women in this population prefer not to lose weight because it is culturally unacceptable, and the fact that losing weight stigmatises them as being HIV positive.

The above challenge supports the importance of considering an individual's integration of cognition, beliefs and/or values and practices, as explained by the indigenous knowledge systems theory.^29^ The system looks at behaviours that occur naturally, regardless of the fact that the individuals are empowered in terms of education (this is also referred to as traditional science). Because of the diversities in beliefs and values in different ethnic groups it is vital to consider each individual's environmental factors.^30^

#### Behaviour change

Patients and professionals perceived behaviour change as a barrier in terms of acceptance, which makes it difficult to control the disease: ‘What are people going to say about me?,’ and ‘How am I going to live with this disease?’, asked participant 4. The overall goal of the management of type 2 diabetes is to help patients and their families gain knowledge, make life skill changes, and offer the support needed to achieve optimal health.^30^ The success of this management requires that health professionals understand the lifestyle, cultural beliefs, attitudes, family and social networks of the patients.^30^ They describe culture as a learned behaviour that is passed from one generation to another, that gives people different attitudes and beliefs.

According to the theory of reasoned action, for successful health education it is important to determine the individual's behavioural intention – which is determined by the patient's attitude towards performing that behaviour and his subjective norm associated with the behaviour.^24^ This means that if patients strongly believe that there is a cure for their disease, it is very difficult to convince them that there is none unless one understands the attitudes and norms that drive that behaviour. Patients with positive beliefs will have positive attitudes towards behavioural change and will be motivated to comply, and those with negative, subjective norms will be less motivated and will resist behaviour change.^30^

For successful type 2 diabetes management individuals should pay more attention to food portions and weight control, as well as engaging in exercises in order to improve their impaired glucose tolerance and fasting glucose. This lifestyle modification will improve their glycaemic control.^9, 30^ Acceptance is the most important way of welcoming change, and patients need to be discouraged from using the information they are given to threaten themselves rather than improving their knowledge: ‘You think of the disease as a stigma’, ‘You think of the complications’, said participant 2.

Challenges are faced when patients compare themselves with others, forgetting that each individual has his or her own limitations and abilities. Patients need to be encouraged always to ‘think out of the box’ in order to discourage them from negative thoughts.^24^ Professionals felt that behaviour change also formed a barrier to disease management: ‘Patients need to cut down their food portions, they also need to exercise; but they need to know what is regarded as sufficient exercise or physical activity and how much is sufficient’, said participant 3.

#### Support

Support is very important in chronic disease management. Patients need emotional support from family and friends: ‘Why me?’ (Participant 4). They also need material support: ‘Where am I going to get money to buy food?’ (Participants 5 & Participant 6). Low socio-economic status makes it difficult to manage the disease. This is because access to and utilisation of medical services, including hospital and nearby health care centres, are related to socio-economic status.^25, 29^

In this study both patients and professionals agreed that changing lifestyle, for example food choices, needs sufficient finances, and most patients struggle to meet the requirements because they lack sufficient finances.

Patients with type 2 diabetes also need informational support. Participant 1 said ‘You think of it as a death sentence especially when you think of the complications’. Support networks give patients strength and a sense of living.^22, 29^

Professionals felt it was important to consider patients’ environmental backgrounds, because these will affect the outcome of the disease management. They emphasised that the relationship of each family with food will affect the individual: ‘Each patient should be assessed individually, families are unique‘, said participant 4; and 'it is very difficult to change what a patient can afford‘, said participants 5. Professionals also felt it important to give information to the wives, relatives or children of each male patient on how to prepare their food, because in African cultures men traditionally do not cook. This is thus a barrier to disease management.

#### Patient-centred approach

In this study the problem of limited time for consultation with the doctor was seen as one of the possible problems that could contribute to patients being non-adherent in their management. 'Patients need more time so that they can ask questions and be asked by the doctor how they feel‘, said participant 9. This was felt more strongly by the professional focus group than by the patient group. Chronic diseases need optimal care; therefore limited time given to patients’ consultations makes providing comprehensive care a challenge.^28^

'A bio-psychosocial approach is important for these patients‘, said participant 3, whilst participants 4, 5 and 7 expressed the view that 'We need to be accommodative, work holistically and work hand in hand with one another as a health team‘. When using a patient-centred approach health care providers can give care that is more effective over time. This approach helps to set goals collaboratively and explores patients’ understanding of the disease and treatment options.

### Limitations

The small sample size and restricted research site limit the transferability of the findings to the general population with type 2 diabetes. However, these findings reflected on challenges that both patients and the health care team experience in managing type 2 diabetes.

### Recommendations

It is recommended that a larger number of focus groups be included from both the rural and urban environment to obtain a cross-sectional representation of quality of care in managing type 2 diabetes in South Africa.

## Conclusion

The findings of this study suggest that patients with type 2 diabetes require reinforcement of knowledge through health communication to encourage them to understand their disease management better, for more appropriate self-care. This will encourage behaviour change through lifestyle modification. A patient-centred approach should be utilised, and each patient's environmental background should be considered.
